# The predictive value of hsCRP/HDL-C ratio for cardiometabolic multimorbidity in middle-aged and elderly people: evidence from a large national cohort study

**DOI:** 10.3389/fnut.2025.1580904

**Published:** 2025-07-10

**Authors:** Shiyang Li, Yuyong Liu, Guangyan Sun, Jie Zhou, Deyun Luo, Guangming Mao, Wenhao Xu

**Affiliations:** ^1^Department of Geriatrics, Panzhihua Central Hospital, Panzhihua, China; ^2^Panzhihua Central Hospital Affiliated to Dali University, Yunnan, China; ^3^Intervention Center, Panzhihua Central Hospital, Panzhihua, China; ^4^Panzhihua Central Hospital, Panzhihua, China

**Keywords:** cardiometabolic multimorbidity, high-sensitive C-reactive protein, high-density lipoprotein cholesterol ratio, CHARLS, risk

## Abstract

**Background:**

Cardiovascular disease is associated with inflammation and dysregulated lipid metabolism. This study aimed to investigate the predictive value of high-sensitive C-reactive protein to high-density lipoprotein cholesterol ratio (CHR) in assessing the risk of developing cardiometabolic multi-morbidity (CMM) within the Chinese population.

**Methods:**

A cohort of 8,187 participants were selected from the China Health and Retirement Longitudinal Study (CHARLS) and divided into four groups based on the quartile of CHR. To evaluate the association between CHR and CMM, we employed multivariable Cox proportional hazards regression, logistic regression, and restricted cubic splines (RCS) analysis. Subgroup analyses and interaction tests were conducted to further explore these relationships.

**Results:**

The mean age of the included participants was 58.64 ± 9.66 years, with 53.7% being female. Over a median follow-up period of 109 months, 858 participants (10.5%) were diagnosed with new-onset CMM. The incidence of CMM across CHR quartiles Q1, Q2, Q3, and Q4 were 6.4, 9.4, 12.0, and 14.2%, respectively. Compared to the lowest quartile, the fully adjusted hazard ratio (with 95% confidence intervals) for CMM for quartiles Q2–Q4 were 1.43 (1.14–1.79), 1.67 (1.35–2.07), and 1.91 (1.55–2.37), respectively. Per 0.01 unit increase in CHR correlates with a 38% increase in the risk of CMM (HR = 1.38, 95% CI = 1.08–1.77, *p* = 0.01) after full adjustment. Additionally, the odds ratios (ORs) (95% CIs) using multivariate logistic regression analysis for participants in quartiles 2 to 4 were 1.47 (1.16–1.86), 1.73 (1.38–2.17), and 2.00 (1.59–2.51), respectively, when compared to participants in Q1 of CHR. Furthermore, a nonlinear relationship was observed between CHR and the risk of CMM (overall *p* < 0.001, nonlinear *p* < 0.001). Subgroup and sensitivity analyses corroborated the robustness of our findings.

**Conclusion:**

A higher CHR was positively associated with the risk of CMM. Our findings suggest that CHR, when considered alongside other risk factors, could serve as a valuable biomarker for identifying individuals at heightened risk of developing CMM.

## Introduction

Cardiometabolic multimorbidity (CMM) was defined as the concurrent presence of at least two cardiometabolic diseases (CMD), including diabetes, heart disease and stroke (1, 2). Individuals with CMM demonstrated a significantly increased risk of mortality and a markedly reduced life expectancy compared to those with single CMD (3). Over recent decades, the prevalence of CMM has increased considerably, attributed to extended life expectancy and various cardiovascular-related risk factors, thereby imposing substantial health and economic burdens on both society and individuals (4, 5). However, existing research has predominantly focused on individual CMDs, often overlooking a comprehensive assessment of this complex condition.

High-sensitive C-reactive protein (hsCRP), an acute-phase protein primarily synthesized by hepatocytes in response to proinflammatory cytokines, serves as a well-established marker of inflammation, capable of detecting minimal concentrations of CRP even in healthy individuals (6, 7). Previous research has demonstrated a strong association between hsCRP and various cardiovascular diseases (CVDs). A cohort study by Lee et al. found that early elevation of serum hsCRP could independently predict the incident CVDs and all-cause mortality (8). In a study involving 387 patients with schizophrenia, an hsCRP level of ≥2.13 mg/L was linked to an increased risk of CVDs (9). Individuals with higher hsCRP exhibited increased risk of developing CMM in a prospective cohort study (10). Conversely, high-density lipoprotein cholesterol (HDL-C) is widely acknowledged as a protective factor against atherosclerosis due to its role in reverse cholesterol transport, antioxidative properties, anti-inflammatory effects, and the protection of vascular endothelial cells (11). An observational study demonstrated an inverse correlation between HDL-C levels and CVDs among participants with HDL-C ≤ 50 mg/dL (12). Similarly, the LUdwigshafen RIsk and Cardiovascular health (LURIC) study identified an inverse relationship between HDL-C and cardiovascular mortality (13). However, due to the heterogeneity of the population and the existence of numerous confounding or residual risk factors, it is difficult to rely solely on hsCRP or lipid levels for prognostic assessment in patients with cardiovascular disease.

Recently, the ratio of hsCRP to HDL-C (CHR) has emerged as an easy-to-assess inflammatory marker associated with various cardiovascular diseases. A study conducted by Gao et al. demonstrated that elevated CHR levels could serve as an independent predictor of CVD, new-onset stroke, and cardiac complications (14). In a prospective study involving 3,260 patients with coronary artery disease following percutaneous coronary intervention, elevated CHR was linked to increased risk of all-cause mortality, cardiac mortality, and major adverse cardiac events (15). Despite these findings, there is a lack of research exploring the association between CHR and the risk of developing CMM among middle-aged and older adults.

In this study, we hypothesized that CHR was correlated with CMM. To test this hypothesis, we conducted a longitudinal analysis utilizing data from the China Health and Retirement Longitudinal Study (CHARLS) to determine the predictive value of CHR for the risk of developing CMM among the Chinese population. This investigation represents the inaugural exploration of the relationship between CHR and CMM in the general Chinese cohort, underscoring the importance of CHR as a significant indicator for CMM in this demographic.

## Methods

### Study design and population

The study utilized data from the China Health and Retirement Longitudinal Study (CHARLS), a national population-based cohort study targeting Chinese adults aged 45 years and older.^1^ This study is designed to promote interdisciplinary aging research and investigate demographic changes in China’s population. Detailed information regarding the study design and participant enrollment criteria has been previously described (16). The study was organized into five survey waves conducted intermittently from 2011 to 2020. Participants were selected using a rigorous multistage stratified probability proportional-to-size sampling technique across 23 provinces in China. The initial national baseline survey was conducted in 2011, followed by four subsequent waves: Wave 2 (2013–2014), Wave 3 (2015–2016), Wave 4 (2017–2018), and Wave 5 (2019–2020).

**Figure 1:**
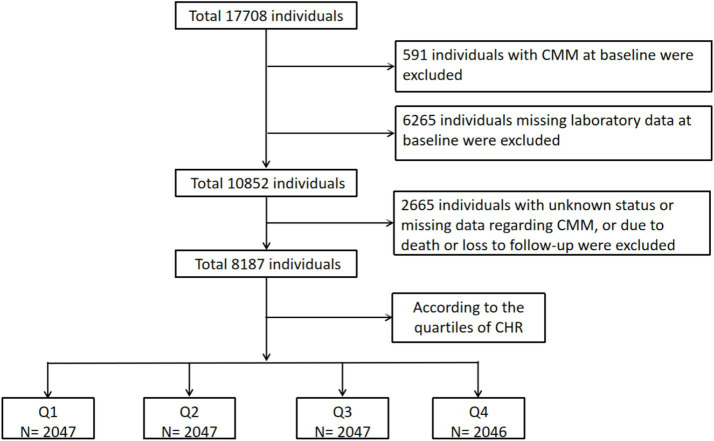
The flowchart of study participants. CMM, cardiometabolic multimorbidity; CHR, ratio of hsCRP to HDL-C.

**Figure 2:**
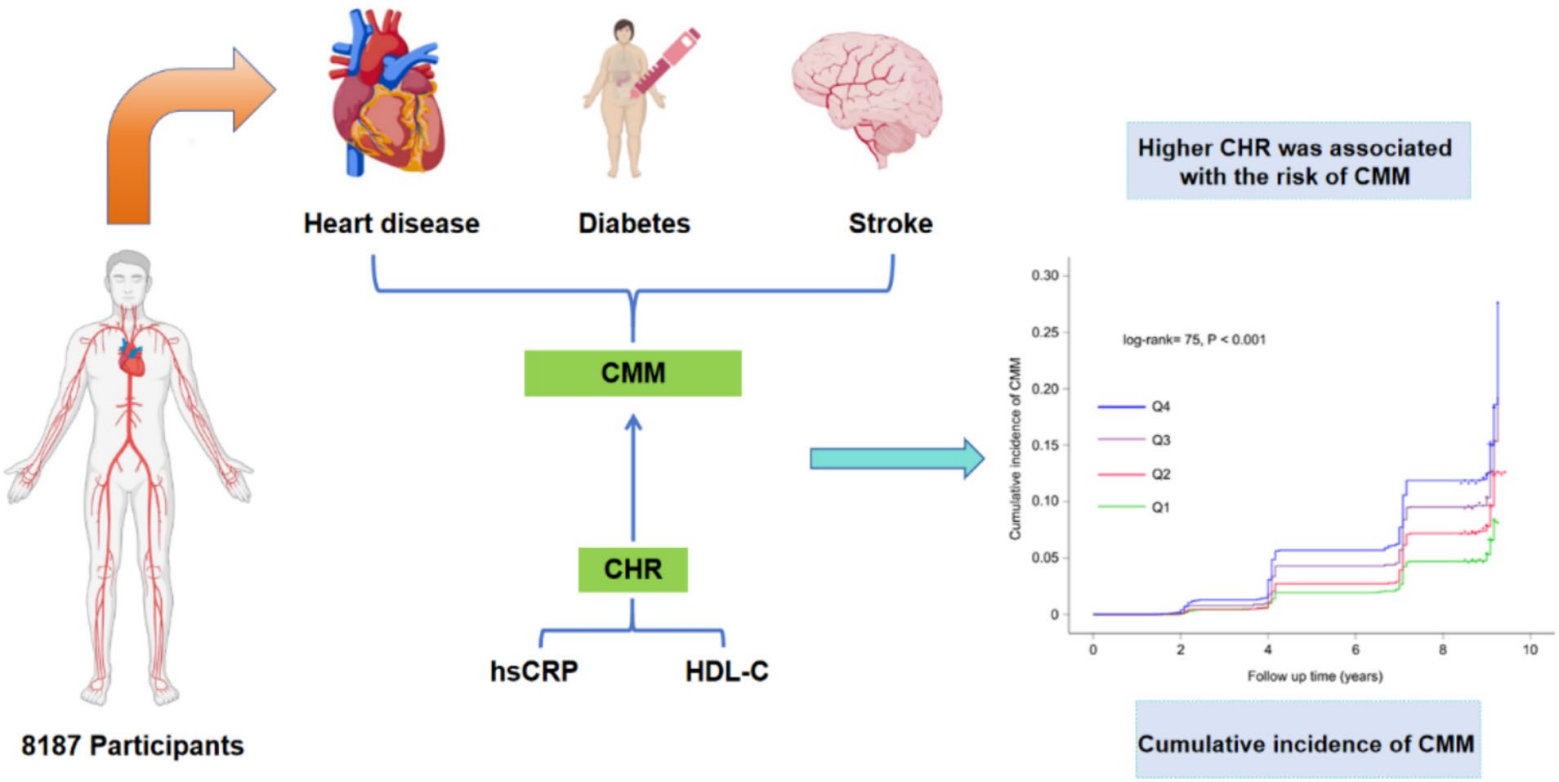
The design flow of this study. CMM, cardiometabolic multimorbidity; CHR, ratio of hsCRP to HDL-C.

Initially, 17,708 participants from the CHARLS were screened, resulting in the selection of 8,187 participants who were categorized into four subgroups based on the quartiles of the CHR index at baseline. The remaining 9,521 individuals were excluded due to the presence of self-reported confirmed CMM at baseline (*N* = 591), missing laboratory data at baseline (*N* = 6,265), unknown status or missing data regarding CMM, or due to death or loss to follow-up (*N* = 2,665). Figure 1 provides a detailed overview of the selection criteria. The design flow of this study is shown in Figure 2. The original CHARLS study received approval from the Ethical Review Committee of Peking University (IRB00001052–11015), and written informed consent was obtained from all participants upon enrollment.

### Assessment of CHR and other covariates

Blood samples were obtained after an overnight fasting period and promptly stored at −20°C to maintain sample integrity before being transported to Beijing for subsequent analysis. The biochemical parameters assessed encompassed a variety of essential health indicators, such as white blood cell count (WBC), platelet count (PLT), hemoglobin, fasting blood glucose (FBG), uric acid (UA), serum creatinine, high-sensitivity C-reactive protein (hsCRP), glycosylated hemoglobin (HbA1c), and lipid profiles. HsCRP levels were quantified utilizing an immunoturbidimetric assay, while HDL-C concentrations were determined through an enzymatic colorimetric test. These analytical procedures were conducted at the Youanmen Center for Clinical Laboratory of Capital Medical University. Estimated glomerular filtration rate (eGFR) was calculated using the CKD-EPI formula (17). In our study, the hsCRP to HDL-C ratio was determined by dividing the hsCRP level (mg/L) by the HDL-C level (mg/dL). Anthropometric measurements, such as height (in meters) and weight (in kilograms), were conducted by qualified medical personnel in accordance with standardized protocols. Body mass index (BMI) was calculated as weight divided by height squared (kg/m^2^). After a minimum of 5 min of seated rest, a trained interviewer measured participants’ blood pressure three times at 45-s intervals using a digital sphygmomanometer (Omron TM HEM-7200).

Information regarding socio-demographic characteristics, lifestyle, and health status were obtained from all participants through structured questionnaires administered during face-to-face interviews. The socio-demographic characteristics included age, sex (male or female), marital status (married and other), residence (rural, urban), and education (elementary school and below, secondary school, and college and above). Lifestyle included smoking and drinking. Health status included hypertension (yes or no), diabetes (yes or no), stroke (yes or no), heart disease (yes or no), and kidney disease (yes or no). Participants were classified as having hypertension if their systolic blood pressure (SBP) was ≥ 140 mmHg, diastolic blood pressure (DBP) was ≥ 90 mmHg, if they self-reported a history of hypertension, or if they were currently taking medications for blood pressure control.

### Assessment of incident CMM

CMM events were characterized by the simultaneous presence of at least two cardiometabolic diseases, such as diabetes, heart disease, and stroke.

The verification of heart disease and stroke diagnoses was based on participants’ self-reported information regarding a physician’s diagnosis, obtained through a questionnaire. This questionnaire included inquiries such as, “Has a doctor ever informed you that you have been diagnosed with a heart attack, angina, coronary heart disease, heart failure, or other heart-related issues?” or “Has a doctor ever informed you that you have been diagnosed with a stroke?” (18). In addition to self-reported diabetes, individuals were classified as diabetic if they met any of the following criteria: (1) fasting plasma glucose levels of ≥ 7.0 mmol/L; (2) random plasma glucose levels of ≥ 11.1 mmol/L; or (3) HbA1c levels of ≥ 6.5%, in accordance with the standards established by the American Diabetes Association (19). The incidence of CMM was determined at the point of diagnosis of the second CMD, at which time individuals exhibited two distinct types of CMD.

### Statistical analysis

Continuous variables were expressed as the mean (standard error) for normally distributed variables and as the median (interquartile range) for variables exhibiting a non-normal distribution. Categorical variables were articulated as frequency (percentage). To assess differences among the groups, one-way analysis of variance was utilized for normally distributed data, Kruskal-Wallis tests for skewed data, and chi-square tests for categorical data. In this study, the cohort was divided into four groups based on the quartiles of baseline CHR: Q1 (< 0.01), Q2 (≥ 0.01, < 0.02), Q3 (≥ 0.02, < 0.05), and Q4 (≥ 0.05). The Kaplan–Meier method was utilized to evaluate the cumulative incidence of CMM across CHR quartiles. Prior to implementing the Cox regression model, the proportional hazards assumption was assessed using Schoenfeld residuals, revealing no significant violations. The Cox proportional hazards regression was then applied to estimate hazard ratios (HRs) and 95% confidence intervals (95% CIs) for the association between CHR quartiles and CMM incidence. Furthermore, logistic regression analyses were conducted to explore the associations between CHR and CMM. The nonlinear relationship between CHR and CMM risk was examined using restricted cubic splines (RCS) analysis. Subgroup analyses were performed to investigate whether the association between CHR and CMM differed according to variables such as sex, age, hypertension, kidney disease, residence, marital status, education, smoking status, and drinking status. The predictive capacity of various variables for CMM was assessed using receiver operating characteristic (ROC) curve analysis.

All statistical analyses in this study were conducted utilizing RStudio version 4.2.1. A threshold for statistical significance was established at bilateral *p*-values of less than 0.05.

## Results

### Baseline characteristics of participants

The mean age of the included participants was 58.64 ± 9.66 years, with 53.7% being female. Generally, individuals with CMM were older, had a higher proportion of females, and demonstrated increased levels of SBP, DBP, BMI, WBC, PLT, hemoglobin, FBG, total cholesterol (TC), triglycerides (TG), low-density lipoprotein cholesterol (LDL-C), UA, hsCRP, HbA1c, and CHR compared to those without CMM (*p* < 0.05) (Table 1). Additionally, this group exhibited a higher prevalence of hypertension, basal diabetes, basal stroke, basal heart disease, and kidney disease (*p* < 0.05) (Table 1). Conversely, individuals with CMM had a lower prevalence of current smoking and drinking, as well as decreased levels of high-density lipoprotein cholesterol (HDL-C) and eGFR (*p* < 0.05).

**Table 1:** Baseline characteristics of the study participants according to cardiometabolic multimorbidity.

Characteristics	Overall (*N* = 8,187)	Non-CMM group (*N* = 7,329)	CMM group (*N* = 858)	*p*-value
CHR	0.02 (0.01–0.05)	0.02 (0.01–0.04)	0.03 (0.01–0.06)	N/A
Age, years	58.64 ± 9.66	58.44 ± 9.78	60.31 ± 8.49	<0.001
Female, n (%)	4,396 (53.69)	3,882 (52.97)	514 (59.91)	<0.001
SBP, mmHg	128.70 ± 21.19	127.82 ± 20.85	136.05 ± 22.53	<0.001
DBP, mmHg	75.01 ± 12.07	74.66 ± 12.01	77.87 ± 12.13	<0.001
Rural residence, n (%)	5,333 (65.14)	4,792 (65.38)	541 (63.05)	0.175
Marriage, married, n (%)	7,252 (88.58)	6,501 (88.70)	751 (87.53)	0.307
Educational level, n (%)				0.21
Primary	5,707 (69.71)	5,087 (69.41)	620 (72.26)	
Secondary	1,656 (20.23)	1,500 (20.47)	156 (18.18)	
Third	824 (10.06)	742 (10.12)	82 (9.56)	
Smoking, n (%)	3,186 (38.92)	2,899 (39.56)	287 (33.45)	<0.001
Current drinking, n (%)	2,742 (33.49)	2,505 (34.18)	237 (27.62)	<0.001
Hypertension, n (%)	3,608 (44.07)	3,037 (41.44)	571 (66.55)	<0.001
Basal diabetes, n (%)	1,043 (12.74)	812 (11.08)	231 (26.92)	<0.001
Basal stroke, n (%)	132 (1.61)	98 (1.34)	34 (3.96)	<0.001
Basal heart disease, n (%)	777 (9.49)	529 (7.22)	248 (28.90)	<0.001
Kidney disease, n (%)	447 (5.46)	368 (5.02)	79 (9.21)	<0.001
BMI, kg/m^2^	23.05 (20.85–25.67)	22.88 (20.70–25.40)	24.96 (22.33–27.55)	<0.001
WBC, 10^9^/L	6.00 (4.95–7.20)	5.90 (4.90–7.20)	6.20 (5.10–7.50)	<0.001
PLT, 10^9^/L	212.35 ± 75.51	211.33 ± 71.90	221.04 ± 100.89	0.006
Hemoglobin, g/dL	14.39 ± 2.23	14.36 ± 2.22	14.61 ± 2.33	0.002
FBG, mg/dL	102.06 (94.32–112.68)	101.52 (93.96–111.42)	108.72 (99.00–123.48)	<0.001
TC, mg/dL	192.95 ± 38.26	192.13 ± 38.02	199.96 ± 39.59	<0.001
TG, mg/dL	104.43 (74.56–153.99)	101.78 (73.46–149.57)	125.67 (87.84–184.96)	<0.001
HDL-C, mg/dL	51.13 ± 15.13	51.57 ± 15.13	47.40 ± 14.69	<0.001
LDL-C, mg/dL	116.40 ± 34.58	115.74 ± 34.08	122.00 ± 38.13	<0.001
UA, mg/dL	4.42 ± 1.25	4.40 ± 1.24	4.55 ± 1.31	0.002
Serum creatinine, mg/dL	0.78 ± 0.24	0.78 ± 0.25	0.79 ± 0.22	0.111
eGFR, mL/min/1.73 m^2^	95.08 (84.42–102.72)	95.49 (84.98–103.13)	92.44 (81.41–99.17)	<0.001
hsCRP, mg/L	0.99 (0.54–2.13)	0.96 (0.52–2.04)	1.34 (0.67–2.56)	<0.001
HbA1c, %	5.10 (4.90–5.40)	5.10 (4.90–5.40)	5.30 (5.00–5.70)	<0.001

SBP, systolic blood pressure; DBP, diastolic blood pressure; BMI, body mass index; WBC, white blood cell count; PLT, platelet count; FBG, fasting blood glucose; TC, total cholesterol; TG, triglycerides; LDL-C, low-density lipoprotein cholesterol; HDL-C, high-density lipoprotein cholesterol; UA, Uric acid; eGFR, estimated glomerular filtration rate; hsCRP, high-sensitivity C-reactive protein; glycosylated hemoglobin, HbA1c; CMM, cardiometabolic multimorbidity; CHR, ratio of hsCRP to HDL-C.

Table 2 displayed the baseline characteristics of participants stratified by CHR quartiles. Compared to Q1, participants in Q2-Q4 were older, more likely to be male, reside in urban areas, exhibited elevated levels of SBP, DBP, BMI, WBC, PLT, hemoglobin, FBG, TG, UA, serum creatinine, hsCRP, and HbA1c (*p* < 0.05), as well as higher prevalence of smoking, hypertension, basal diabetes, basal stroke, basal heart disease. Conversely, the level of HDL-C and eGFR were lower among participants in the higher CHR quartiles (*p* < 0.05).

**Table 2:** Characteristics of 8,187 participants according to the quartiles of CHR.

Characteristics	CHR				
	Q1 (< 0.01)	Q2 (≥ 0.01, < 0.02)	Q3 (≥ 0.02, < 0.05)	Q4 (≥ 0.05)	*P*-value
Participants, No	2047	2047	2047	2046	
CMM, n (%)	131 (6.40)	192 (9.38)	245 (11.97)	290 (14.17)	N/A
Age, years	57.27 ± 9.64	58.34 ± 9.50	58.93 ± 9.45	60.01 ± 9.87	<0.001
Female, n (%)	1,192 (58.23)	1,103 (53.88)	1,058 (51.69)	1,043 (50.98)	<0.001
SBP, mmHg	125.70 ± 21.12	126.73 ± 20.33	130.06 ± 21.11	132.33 ± 21.53	<0.001
DBP, mmHg	73.25 ± 11.98	74.31 ± 11.93	75.83 ± 12.08	76.65 ± 12.00	<0.001
Rural residence, n (%)	1,455 (71.08)	1,326 (64.78)	1,299 (63.46)	1,253 (61.24)	<0.001
Marriage, married, n (%)	1832 (89.50)	1825 (89.15)	1814 (88.62)	1781 (87.05)	0.068
Educational level, n (%)					0.442
Primary	1,462 (71.42)	1,430 (69.86)	1,400 (68.39)	1,415 (69.16)	
Secondary	398 (19.44)	413 (20.18)	423 (20.66)	422 (20.63)	
Third	187 (9.14)	204 (9.97)	224 (10.94)	209 (10.22)	
Smoking, n (%)	699 (34.15)	776 (37.91)	836 (40.84)	875 (42.77)	<0.001
Current drinking, n (%)	710 (34.68)	726 (35.47)	665 (32.49)	641 (31.33)	0.018
Hypertension, n (%)	751 (36.69)	809 (39.52)	984 (48.07)	1,064 (52.00)	<0.001
Basal diabetes, n (%)	162 (7.91)	217 (10.60)	283 (13.83)	381 (18.62)	<0.001
Basal stroke, n (%)	25 (1.22)	23 (1.12)	32 (1.56)	52 (2.54)	<0.001
Basal heart disease, n (%)	157 (7.67)	174 (8.50)	227 (11.09)	219 (10.70)	<0.001
Kidney disease, n (%)	119 (5.81)	106 (5.18)	113 (5.52)	109 (5.33)	0.827
BMI, kg/m^2^	21.72 (19.91–23.68)	22.89 (20.92–25.29)	23.88 (21.56–26.40)	24.13 (21.58–27.07)	<0.001
WBC, 10^9^/L	5.50 (4.60–6.60)	5.80 (4.80–6.90)	6.04 (5.10–7.30)	6.69 (5.50–8.00)	<0.001
PLT, 10^9^/L	208.32 ± 66.22	208.38 ± 74.34	214.78 ± 83.23	217.92 ± 76.86	<0.001
Hemoglobin, g/dL	14.10 ± 2.22	14.36 ± 2.08	14.62 ± 2.22	14.47 ± 2.38	<0.001
FBG, mg/dL	100.08 (92.70–108.00)	101.52 (94.32–111.46)	102.60 (94.86–113.04)	104.76 (96.12–118.26)	<0.001
TC, mg/dL	193.00 ± 35.29	191.74 ± 36.52	195.28 ± 38.33	191.77 ± 42.42	0.009
TG, mg/dL	84.96 (64.61–115.93)	100.89 (74.34–143.37)	116.82 (83.19–170.80)	123.01 (83.19–189.39)	<0.001
HDL-C, mg/dL	60.72 ± 15.42	52.10 ± 13.17	47.84 ± 13.06	43.85 ± 13.32	<0.001
LDL-C, mg/dL	114.39 ± 31.51	117.58 ± 32.74	119.46 ± 36.02	114.15 ± 37.46	<0.001
UA, mg/dL	4.07 ± 1.11	4.31 ± 1.19	4.57 ± 1.25	4.71 ± 1.32	<0.001
Serum creatinine, mg/dL	0.75 ± 0.18	0.77 ± 0.18	0.79 ± 0.22	0.81 ± 0.36	<0.001
eGFR, mL/min/1.73 m^2^	96.87 (87.96–104.31)	95.57 (85.43–102.95)	93.88 (82.64–101.76)	93.49 (81.81–101.82)	<0.001
hsCRP, mg/L	0.38 (0.29–0.48)	0.73 (0.59–0.89)	1.38 (1.12–1.77)	3.87 (2.61–6.94)	<0.001
HbA1c, %	5.10 (4.80–5.30)	5.10 (4.90–5.40)	5.10 (4.90–5.40)	5.20 (4.90–5.50)	<0.001

SBP, systolic blood pressure; DBP, diastolic blood pressure; BMI, body mass index; WBC, white blood cell count; PLT, platelet count; FBG, fasting blood glucose; TC, total cholesterol; TG, triglycerides; LDL-C, low-density lipoprotein cholesterol; HDL-C, high-density lipoprotein cholesterol; UA, Uric acid; eGFR, estimated glomerular filtration rate; hsCRP, high-sensitivity C-reactive protein; glycosylated hemoglobin, HbA1c; CMM, cardiometabolic multimorbidity; CHR, ratio of hsCRP to HDL-C.

Additionally, we conducted a comparative analysis of the baseline characteristics between included participants and those lacking baseline laboratory data. As demonstrated in Supplementary Table S1, participants missing baseline laboratory data were more likely to be male and reside in urban areas, less likely to be married, and tended to have attained higher levels of education. Additionally, they exhibited a higher prevalence of smoking (*p* < 0.05). Conversely, this group showed a lower prevalence of hypertension, baseline diabetes, baseline heart disease, and kidney disease (*p* < 0.05).

### Relationship of CHR with the incidence of CMM

**Figure 3:**
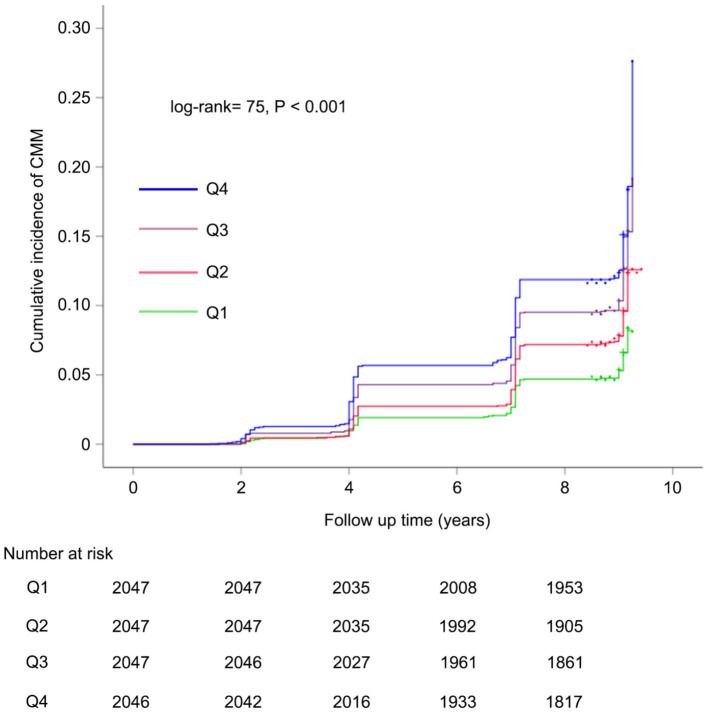
Kaplan–Meier curves for the cumulative incidence of CMM. Q1 is CHR < 0.01; Q2 is CHR ≥ 0.01 but < 0.02; Q3 is CHR ≥ 0.02 but< 0.05; Q4 is CHR ≥ 0.05. CMM, cardiometabolic multimorbidity; CHR, ratio of hsCRP to HDL-C.

Over a median follow-up duration of 109 months, a total of 858 participants (10.5%) were diagnosed with new-onset CMM. The incidence of CMM across CHR quartiles Q1, Q2, Q3, and Q4 were 6.4, 9.4, 12.0, and 14.2%, respectively. As illustrated in Figure 3, there was a progressive increase in the cumulative incidence of CMM corresponding with higher CHR quartiles (log-rank = 75, *p* < 0.001). Table 3 presents the incidence and HR with 95% CI of CHR for CMM. Per 0.01 unit increase in CHR was associated with an elevated risk of incident CMM in the unadjusted Cox proportional hazards model (model 1) (HR = 1.41, 95% CI = 1.13–1.76). This association remained statistically significant (HR = 1.38, 95% CI = 1.08–1.77) after adjusting for multiple covariates (Model 3), including age, sex, hypertension, smoking status, drinking status, rural residence, marital status, education, WBC, PLT, eGFR, and hemoglobin. Consistent with these findings, Model 3 indicated that the fully adjusted HRs (95% CIs) for participants in CHR quartiles Q2, Q3 and Q4 were 1.43 (1.14–1.79), 1.67 (1.35–2.07) and 1.91 (1.55–2.37), respectively, compared with those in Q1.

**Table 3:** Cox proportional hazards regression model analysis of baseline CHR and CMM.

Variable	Model 1		Model 2		Model 3	
	HR (95%CI)	*P*-value	HR (95%CI)	*P*-value	HR (95%CI)	*P*-value
CHR, continuous	1.41 (1.13–1.76)	0.002	1.42 (1.13–1.79)	0.003	1.38 (1.08–1.77)	0.01
CHR, categories						
Q1	Reference		Reference		Reference	
Q2	1.48 (1.19–1.85)	<0.001	1.49 (1.19–1.86)	<0.001	1.43 (1.14–1.79)	0.002
Q3	1.92 (1.55–2.38)	<0.001	1.92 (1.55–2.38)	<0.001	1.67 (1.35–2.07)	<0.001
Q4	2.32 (1.89–2.85)	<0.001	2.27 (1.85–2.80)	<0.001	1.91 (1.55–2.37)	<0.001

CMM, cardiometabolic multimorbidity; CHR, ratio of hsCRP to HDL-C; HR, hazard ratio; CI, confidence interval; eGFR, estimated glomerular filtration rate. Model 1: unadjusted. Model 2: adjusted for age, sex. Model 3: model 2 + further adjusted for hypertension, smoking status, drinking status, rural residence, marital status, education, WBC, PLT, eGFR, and hemoglobin.

We performed logistic regression analysis to further substantiate the association between baseline CHR and the risk of CMM. As a continuous variable, per 0.01 unit increase in the CHR was correlated with a 45% increase in the risk of CMM (OR = 1.45, 95% CI = 1.06–1.98, *p* = 0.018) after full adjustment (Table 4). As a categorical variable, individuals in quartiles Q2, Q3, and Q4 exhibited a significantly increased risk of CMM with multi-adjusted logistic regression analysis (Q2 vs. Q1: OR = 1.47, *p* = 0.001 in model 3; Q3 vs. Q1: OR = 1.73, *p* < 0.001 in model 3; and Q4 vs. Q1: OR = 2.00, *p* < 0.001 in model 3).

**Table 4:** Logistic regression analysis of baseline CHR and CMM.

Variable	Model 1		Model 2		Model 3	
	OR (95%CI)	*P*-value	OR (95%CI)	*P*-value	OR (95%CI)	*P*-value
CHR, continuous	1.49 (1.13–1.96)	0.005	1.50 (1.12–1.99)	0.006	1.45 (1.06–1.98)	0.018
CHR, categories						
Q1	Reference		Reference		Reference	
Q2	1.51 (1.20–1.91)	<0.001	1.52 (1.21–1.92)	<0.001	1.47 (1.16–1.86)	0.001
Q3	1.99 (1.59–2.48)	<0.001	1.99 (1.59–2.49)	<0.001	1.73 (1.38–2.17)	<0.001
Q4	2.42 (1.95–3.00)	<0.001	2.37 (1.91–2.95)	<0.001	2.00 (1.59–2.51)	<0.001

CMM, cardiometabolic multimorbidity; CHR, ratio of hsCRP to HDL-C; OR, odds ratio; CI, confidence interval; eGFR, estimated glomerular filtration rate. Model 1: unadjusted. Model 2: adjusted for age, gender. Model 3: model 2 + further adjusted for hypertension, smoking status, drinking status, rural residence, marital status, education, WBC, PLT, eGFR, and hemoglobin.

**Figure 4:**
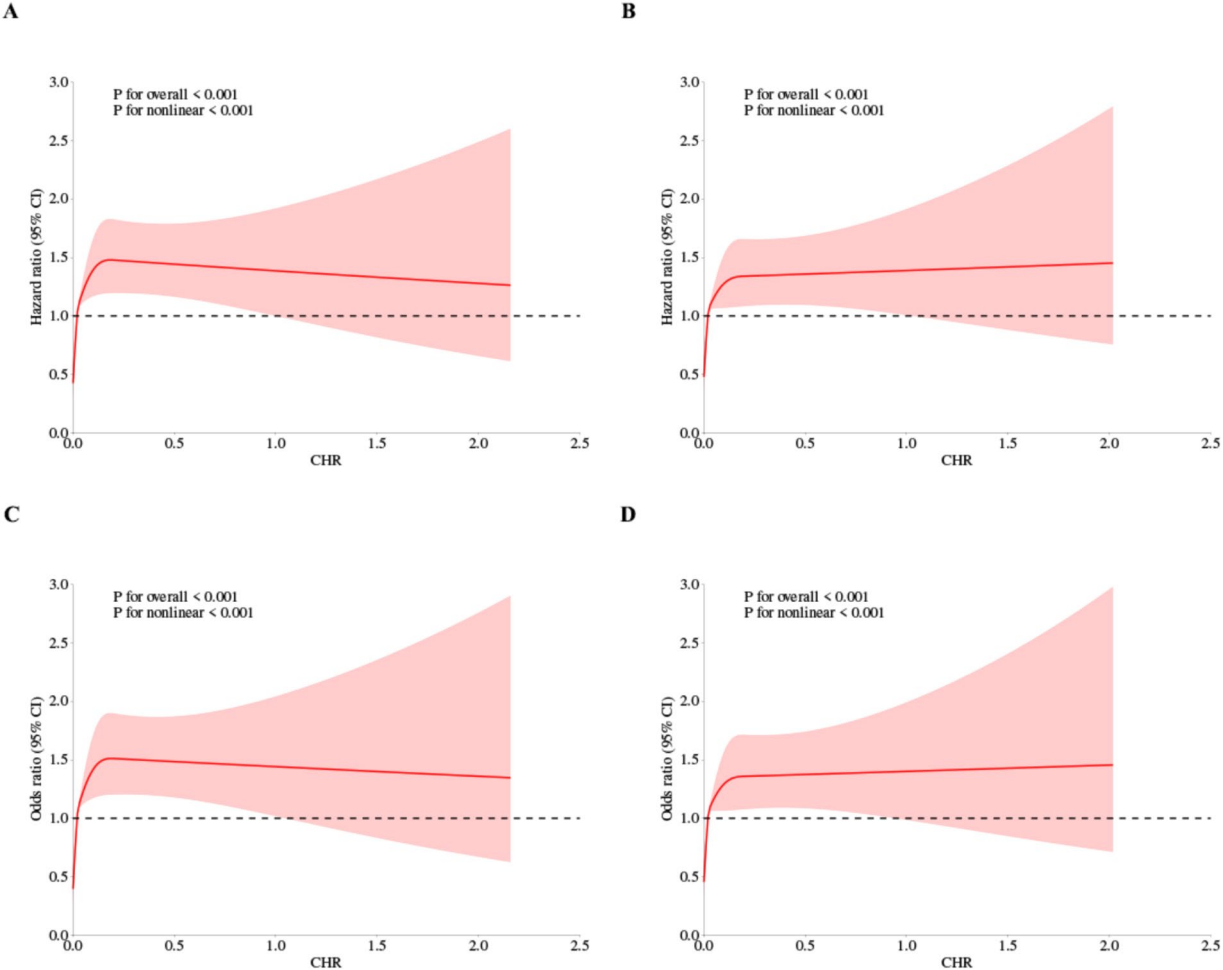
Restricted cubic spline curves for CMM by CHR using Cox proportional hazards regression model analysis **(A,B)** and logistic regression analysis **(C,D)**, without **(A,C)** or with **(B,D)** adjustment for other covariates including age, sex, hypertension, kidney disease, smoking status, drinking status, rural residence, marital status, education.

Furthermore, a 4-knot RCS regression model was utilized to precisely characterize the dose–response curves of CHR in relation to the risk of CMM. Figure 4 illustrates a significant nonlinear dose–response relationship between CHR and the incidence of CMM, as determined by both Cox proportional hazards regression and logistic regression analyses (P for overall trend < 0.001; P for nonlinear trend < 0.001).

### Subgroup analyses

**Figure 5:**
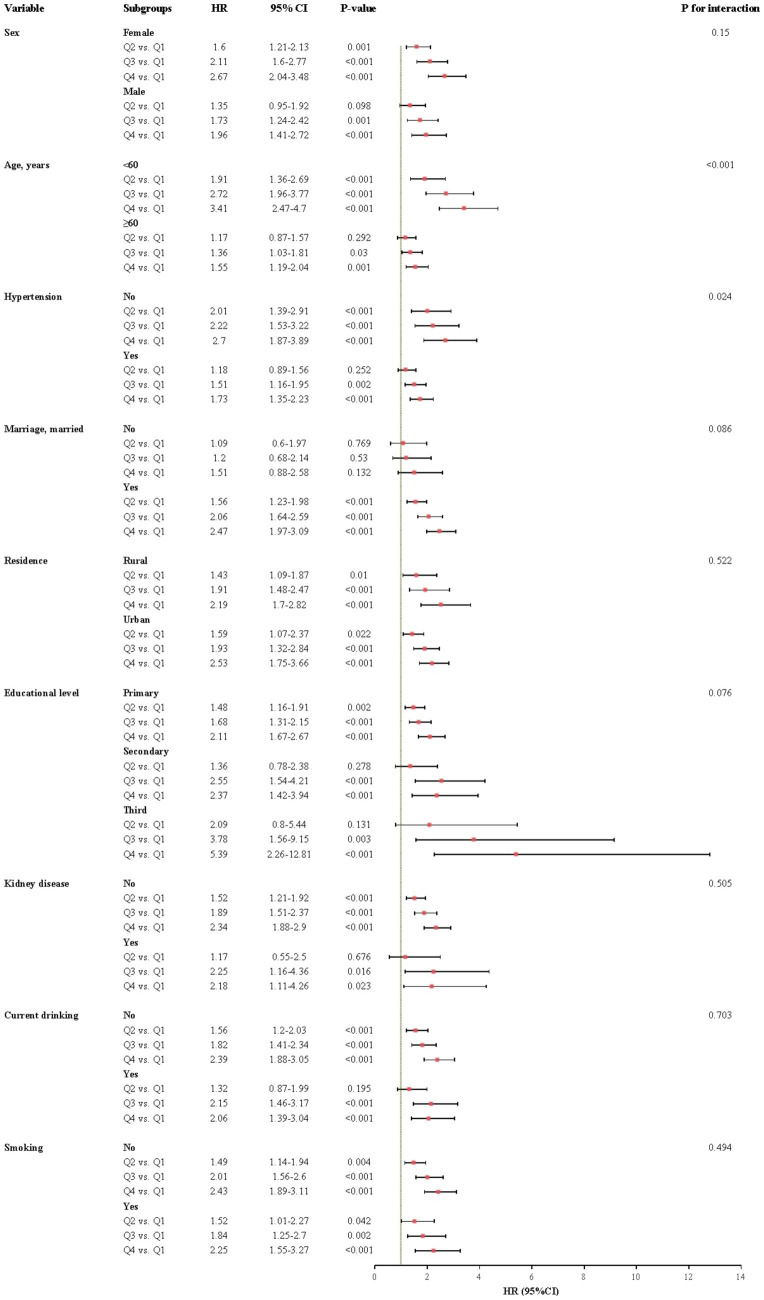
Subgroup and interaction analyses between the CHR and CMM across various subgroups using cox proportional hazards regression model. CMM, cardiometabolic multimorbidity; HR, hazard ratios; CI, confidence interval.

**Figure 6:**
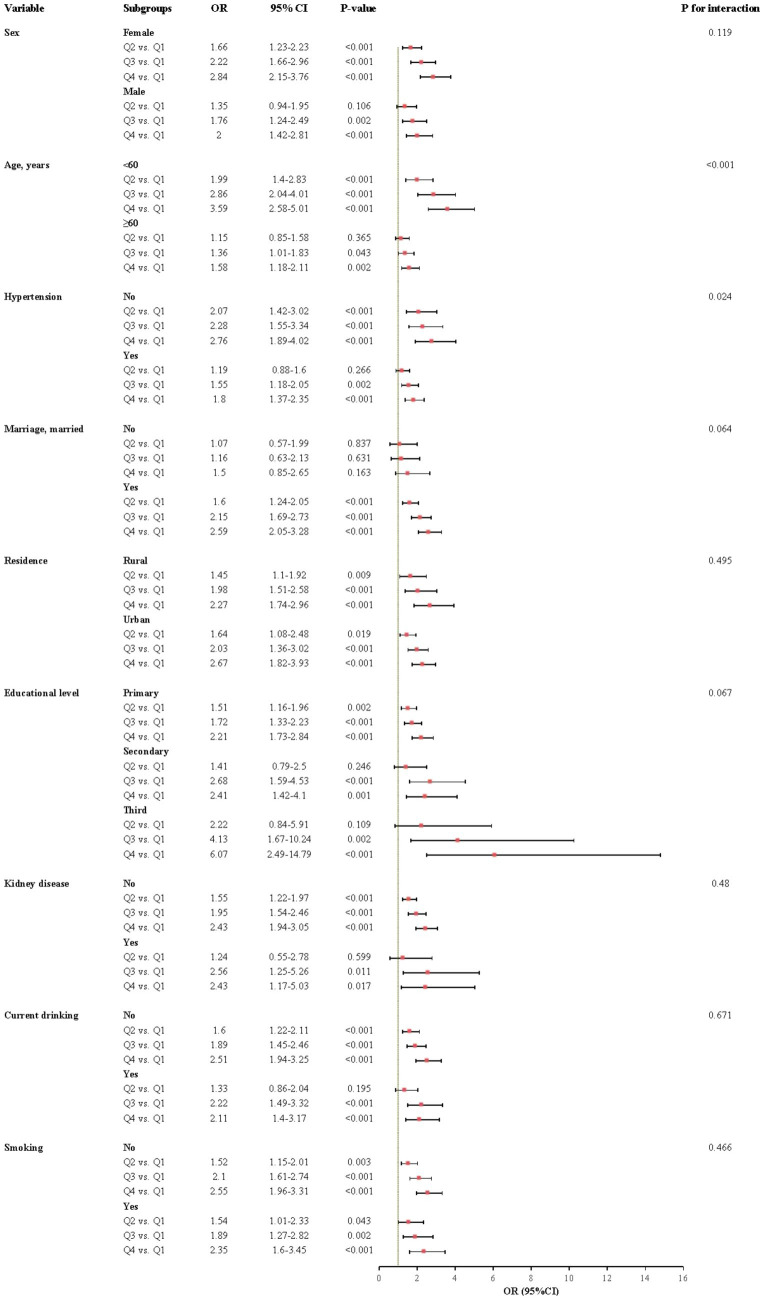
Subgroup and interaction analyses between the CHR and CMM across various subgroups using logistic regression model. CMM, cardiometabolic multimorbidity; OR, odds ratio; CI, confidence interval.

The relationship between CHR and the incidence of CMM was further evaluated across various subgroups. Our results indicated that the association between CHR and CMM was consistent across most subgroups, as depicted in Figures 5, 6. Importantly, age and hypertension were found to significantly influence the association between CHR and the risk of CMM (all P for interaction < 0.05). Furthermore, the population was stratified into two groups based on eGFR values. As demonstrated in Table 5, individuals in the Q4 quartile exhibited a consistently higher risk of CMM in both eGFR subgroups (<90 and ≥90 mL/min/1.73 m^2^) compared to those in the Q1 quartile. Additionally, the population was evenly divided into four age-based groups. The analysis revealed that the association between CHR and CMM remained consistent across the first three age quartiles, while it was not observed in the fourth age quartile (Table 5).

**Table 5:** Subgroup analysis stratified by eGFR.

Subgroup	CHR, categories	Cox proportional hazards regression analysis		Logistic regression analysis	
		HR (95%CI)	*P*-value	OR (95%CI)	*P*-value
eGFR, mL/min/1.73 m^2^					
<90 (*N* = 2,951)	Q1	Reference		Reference	
	Q2	1.23 (0.86–1.74)	0.261	1.23 (0.85–1.78)	0.283
	Q3	1.40 (1.00–1.95)	0.049	1.42 (1.00–2.01)	0.053
	Q4	2.16 (1.58–2.95)	<0.001	2.26 (1.62–3.16)	<0.001
≥90 (*N* = 5,236)	Q1	Reference		Reference	
	Q2	1.64 (1.23–2.18)	<0.001	1.68 (1.25–2.26)	<0.001
	Q3	2.27 (1.73–2.99)	<0.001	2.38 (1.78–3.16)	<0.001
	Q4	2.22 (1.68–2.92)	<0.001	2.30 (1.73–3.07)	<0.001
Age, years					
Quartiles 1 (*N* = 2,046)	Q1	Reference		Reference	
	Q2	1.69 (0.96–2.98)	0.068	1.74 (0.97–3.09)	0.061
	Q3	2.82 (1.66–4.78)	<0.001	2.95 (1.72–5.08)	<0.001
	Q4	2.96 (1.73–5.06)	<0.001	3.06 (1.76–5.32)	<0.001
Quartiles 2 (*N* = 2,047)	Q1	Reference		Reference	
	Q2	2.19 (1.33–3.62)	0.002	2.33 (1.39–3.91)	0.001
	Q3	2.82 (1.73–4.58)	<0.001	2.99 (1.80–4.94)	<0.001
	Q4	3.65 (2.26–5.88)	<0.001	3.90 (2.38–6.40)	<0.001
Quartiles 3 (*N* = 2047)	Q1	Reference		Reference	
	Q2	1.35 (0.91–2.00)	0.133	1.32 (0.88–2.00)	0.185
	Q3	1.69 (1.16–2.47)	0.006	1.72 (1.16–2.57)	0.008
	Q4	2.27 (1.59–3.26)	<0.001	2.36 (1.61–3.47)	<0.001
Quartiles 4 (*N* = 2,047)	Q1	Reference		Reference	
	Q2	1.00 (0.67–1.48)	0.982	0.99 (0.65–1.50)	0.949
	Q3	1.09 (0.75–1.59)	0.638	1.09 (0.73–1.62)	0.688
	Q4	1.14 (0.80–1.64)	0.469	1.14 (0.77–1.67)	0.512

### Sensitivity analyses

To enhance the robustness of our findings, several sensitivity analyses were conducted in the present study. First, we excluded participants with heart disease at baseline (*N* = 777), and the association between CHR and CMM remained consistent (Q2 vs. Q1: HR = 1.52, 95% CI = 1.17–1.98; Q3 vs. Q1: HR = 1.74, 95% CI = 1.35–2.25; Q4 vs. Q1: HR = 2.08, 95% CI = 1.62–2.68; for each 0.01-unit increase, HR = 1.39, 95% CI = 1.05–1.83) (Supplementary Table S1). Subsequently, we excluded participants with diabetes at baseline (N = 1,043), and observed no significant alteration in the association (Q2 vs. Q1: HR = 1.39, 95% CI = 1.08–1.80; Q3 vs. Q1: HR = 1.69, 95% CI = 1.32–2.15; Q4 vs. Q1: HR = 1.93, 95% CI = 1.51–2.47) (Supplementary Table S2). A similar relationship was observed when we excluded participants with stroke at baseline (N = 132) (Q2 vs. Q1: HR = 1.45, 95% CI = 1.16–1.82; Q3 vs. Q1: HR = 1.72, 95% CI = 1.38–2.14; Q4 vs. Q1: HR = 1.89, 95% CI = 1.52–2.35; for each 0.01-unit increase, HR = 1.38, 95% CI = 1.07–1.78) (Supplementary Table S2). Finally, we excluded participants who had experienced either heart disease, diabetes, or stroke at baseline (N = 1952), and the results were similar to those of the primary analysis (Q2 vs. Q1: HR = 1.47, 95% CI = 1.06–2.04; Q3 vs. Q1: HR = 1.68, 95% CI = 1.22–2.33; Q4 vs. Q1: HR = 1.78, 95% CI = 1.28–2.47) (Supplementary Table S2).

Furthermore, we conducted logistic regression analysis and observed the similar results (Supplementary Table S3).

### ROC analysis

**Figure 7:**
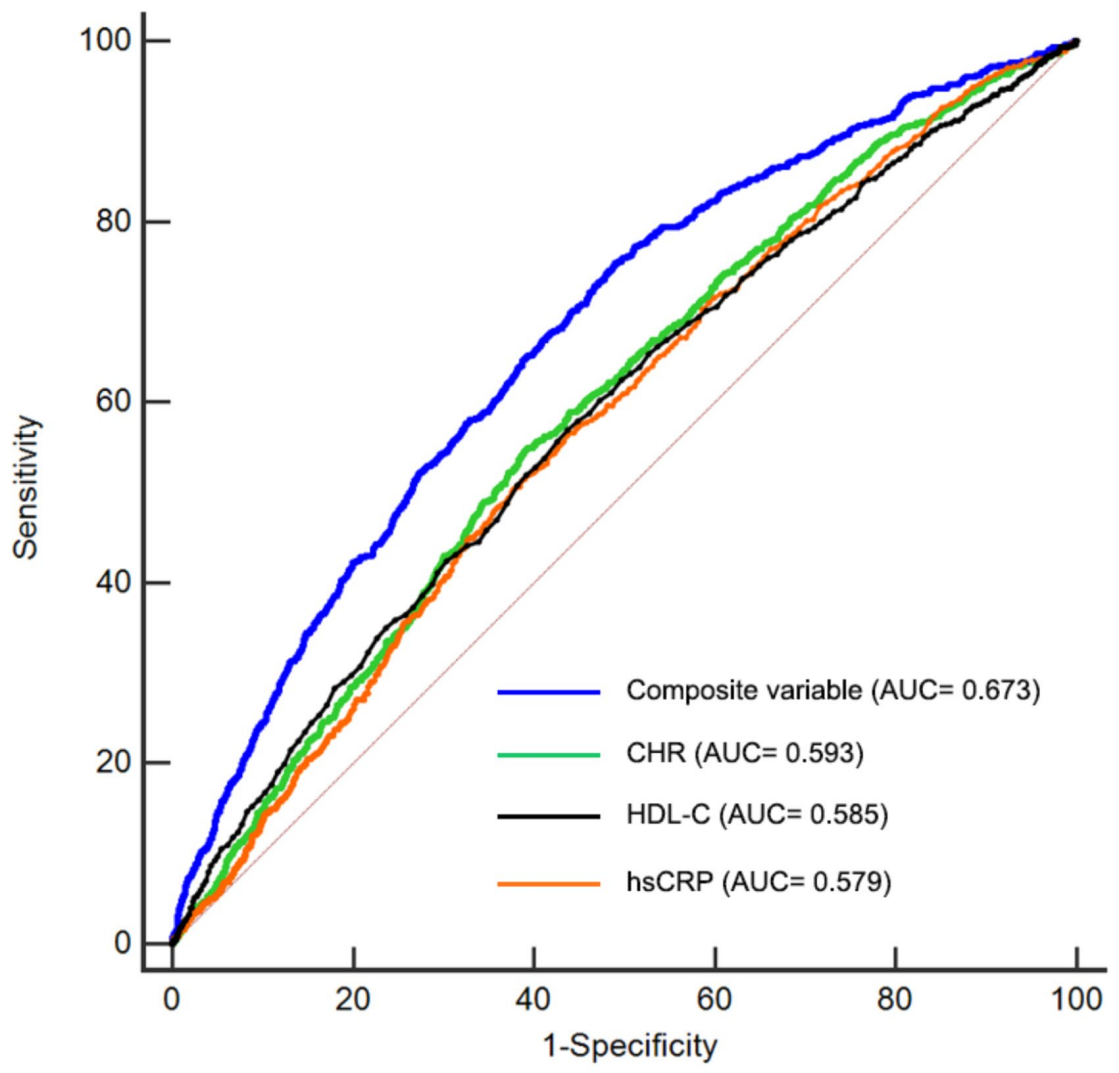
Receiver-operating characteristic curves for prediction of CMM. HDL-C, high-density lipoprotein cholesterol; hsCRP, high-sensitivity C-reactive protein; CHR, ratio of hsCRP to HDL-C; CMM, cardiometabolic multimorbidity; AUC, area under curve.

In this study, we performed ROC analysis to evaluate the predictive capabilities of CHR, hsCRP, HDL-C, and the composite variable (CHR, hypertension, kidney disease, smoking status, drinking status, rural residence, marital status, education, WBC, PLT, and hemoglobin) concerning the risk of CMM. As illustrated in Figure 7 and Table 6, the average AUCs for CHR, hsCRP, HDL-C, and the composite variable were 0.593 (95% CI = 0.582–0.604), 0.579 (95% CI = 0.568–0.589), 0.585 (95% CI = 0.574–0.596), and 0.673 (95% CI = 0.663–0.683), respectively. It is noteworthy that CHR exhibited significantly higher AUC values for predicting CMM risk compared to hsCRP (*p* < 0.001). Although the difference between CHR and HDL-C did not reach statistical significance (*p* = 0.44), the observed trend suggests that CHR may provide a more comprehensive assessment of CMM risk when considering the combined effects of HDL-C and hsCRP. Importantly, the composite variable demonstrated the highest predictive power (p < 0.001).

**Table 6:** ROC analysis.

Variables	AUC	95% CI	Cutoff-value	Specificity	Sensitivity
hsCRP	0.579	0.568–0.589	1.325	0.62	0.51
HDL-C	0.585	0.574–0.596	48.132	0.55	0.58
CHR	0.593	0.582–0.604	0.027	0.61	0.55
Composite variables	0.673	0.663–0.683	0.08	0.51	0.76

ROC, receiver operating characteristic; hsCRP, high-sensitivity C-reactive protein; HDL-C, high-density lipoprotein cholesterol; CHR, ratio of hsCRP to HDL-C; AUC, area under curve; CI, Confidence interval.

## Discussion

We performed a comprehensive longitudinal analysis utilizing CHARLS datasets to evaluate the association between CHR and the risk of CMM. Our findings demonstrated that a higher CHR was correlated with an increased risk of CMM. RCS analyses further validated a positive and nonlinear association between CHR and the risk of CMM, even after adjusting for potential confounders. Additionally, CHR offered a more comprehensive evaluation of CMM risk compared to the separate assessment of HDL-C and hsCRP. Nonetheless, CHR demonstrated only moderate discriminatory power for CMM. There was no statistically significant difference in the predictive power of CHR and HDL-C for CMM. Additionally, integrating CHR with other risk factors substantially improved the predictive capability for the risk of developing CMM.

High-sensitivity C-reactive protein (hsCRP) is an acute-phase protein synthesized by hepatic aortic endothelial cells and coronary artery smooth muscle cells in response to proinflammatory cytokines. Previous clinical studies have established a strong association between hsCRP levels and CVDs. A nationwide prospective cohort study conducted by Xu et al. identified a correlation between elevated hsCRP levels and an increased risk of CMM (10). A systematic review indicated that hsCRP was linked to the incidence of HF in both general and high-risk populations (20). In a cross-sectional study involving 225 asymptomatic diabetic patients, hsCRP was demonstrated to be associated with silent myocardial ischemia (21). In contrast, HDL-C exhibits anti-oxidant, anti-inflammatory, anti-apoptotic, and antithrombotic properties (22). A substantial body of research has documented the protective effects of HDL-C against CVDs (23, 24). However, due to population heterogeneity and the presence of various confounding or residual risk factors, it remains challenging to rely solely on hsCRP or lipid levels for assessing CVD risk (25–27).

The ratio of hsCRP to HDL-C (CHR), which integrates both the risk factor (hsCRP) and the protective factor (HDL-C) in CVDs, has emerged as a significant indicator of inflammation and lipid disorders (14). A retrospective cohort study identified CHR as an independent risk factor for both long-term all-cause mortality and cardiovascular mortality in the general population (28). A study conducted by Gao et al. demonstrated that CHR could independently predict CVD, new stroke, and heart problems (14). In a prospective study involving 3,260 patients with coronary artery disease following percutaneous coronary intervention, elevated CHR was linked to increased risk of all-cause mortality, cardiac mortality, and major adverse cardiac events (15). Furthermore, in heart failure patients with preserved ejection fraction, HDL-C/CRP ratio was found to be a valuable marker for predicting all-cause death and cardiac death and correlated with left ventricular diastolic function and right ventricular systolic function (29). In the present study, we also observed the similar results that higher CHR was associated with increased risk of CMM among middle-aged and elderly populations in China, corroborating the findings of previous research. Notably, investigations into the predictive value of CHR for CMM risk have been limited, as prior studies have predominantly focused on individual CMDs. This study is the first to examine the association between CHR and the risk of developing CMM utilizing a nationally representative prospective cohort.

Cardiometabolic multimorbidity (CMM) is defined as the concurrent presence of a minimum of two cardiometabolic conditions, including heart disease, diabetes, and stroke (30). Previous research has identified numerous risk factors associated with CMM. For instance, a cohort study by Lu et al. demonstrated that waist-to-height ratio, waist circumference, waist divided by height^0.5^, and BMI were all independent predictors of CMM (31). However, the cross-sectional design of their study limited the ability to establish causality. In contrast, the prospective design of our study enhances the robustness and reliability of the findings. Behnam et al. identified elevated triglyceride, VLDL-C, total cholesterol/HDL-C, TG/HDL-C, and apoB/apoA1 as risk factors for CMM (32). Their study was limited to 1,728 male participants. Instead, our study encompassed a larger population that included both males and females, thereby enhancing the robustness and generalizability of our findings. Additionally, a study by Guo et al. identified impaired fasting glucose as a risk factor for CMM (33). However, their exclusion of participants with diabetes mellitus and cardiovascular disease at baseline likely restricts the applicability of their conclusions to the broader population. Our research demonstrated that CHR is associated with the risk of CMM in the general population, regardless of diabetes mellitus and cardiovascular disease status. Furthermore, Liu et al. identified both the visceral adiposity index and handgrip strength as the predictors of CMM (34). However, they did not perform sensitivity analyses based on different types of CMDs, which could have yielded more nuanced insights. Our sensitivity analysis revealed a consistent association between CHR and CMM in populations excluding heart disease only, excluding stroke only, excluding diabetes only, or excluding any CMD at baseline.

The potential mechanisms underlying the association between CHR and CMM remain poorly understood, but may involve several biological processes. HDL-C plays a crucial role in reverse cholesterol transport and exhibits a range of protective effects, including anti-inflammatory, antioxidative, antithrombotic, and cytoprotective properties (35). Notably, the anti-inflammatory capacity of HDL-C impedes the oxidative modification of LDL-C by reducing the expression of adhesion molecules on endothelial cell surfaces (36). Additionally, HDL-C inhibits the adhesion and migration of T lymphocytes and monocytes to the vascular endothelium and atherosclerotic sites (37, 38). Conversely, inflammatory processes lead to a decrease in HDL-C levels and alterations in HDL-C structure, along with significant modifications in HDL-C-associated proteins, enzymes, and transfer proteins that are integral to HDL-C metabolism and function (39). Owing to the interplay between reduced levels of HDL-C and elevated hsCRP, a comprehensive CHR indicator may offer a more effective and reliable measure of inflammation levels compared to a singular indicator.

It is noteworthy that the association between CHR and the risk of CMM was significantly influenced by age and hypertension in our study. Individuals with age≥ 60 years exhibited higher incidence of hypertension, basal diabetes, basal stroke, and basal heart disease (*p* < 0.05) compared to those with age< 60 years in our study. And these older people are more likely to receive pharmacological treatments, such as beta blockers, angiotensin-converting enzyme inhibitors/angiotensin receptor blockers, glucose-lowering medications, and lipid-lowering medications, which may mitigate the progression of CVDs and contribute to a lower hazard ratio among older people. Similarly, the increased prevalence of baseline diabetes, baseline stroke, and baseline heart disease among participants with hypertension was associated with greater medication use, potentially explaining the lower hazard ratios observed in the hypertensive group compared to the non-hypertensive group in our study.

The present study employs data from the CHARLS, a large-scale and nationally representative prospective cohort. Our study effectively controlled for confounding variables and assessed the nonlinear association between CHR and the risk of CMM using RCS analysis, thereby enhancing the credibility of the findings. Nevertheless, there are certain limitations associated with this study. First, the study primarily focused on individuals aged ≥ 45 years in China, which may limit the generalizability of the results to younger populations and individuals from diverse racial backgrounds. Consequently, further research involving participants from various ethnicities and countries is necessary to evaluate the generalizability of these findings. Second, the analysis was restricted to baseline CHR levels, without accounting for potential fluctuations and dynamic changes over time that could offer valuable insights into the underlying mechanisms. Third, CMM was diagnosed based on self-reported physician assessments, which may introduce potential information bias. To substantiate our findings, future research should involve large-scale, randomized controlled trials. Forth, although numerous potential confounding factors have been adjusted for, the possibility of residual confounding cannot be completely eliminated. Fifth, as an observational study, we cannot establish a causal relationship between CHR and the risk of CMM. Fifth, excluding a large number of participants due to missing baseline laboratory data could introduce the selection bias. But ultimately, our study included a large enough population to mitigate the effects of selection bias to some extent. Lastly, the extended data collection period and the absence of medication data from participants may have influenced the results. For instance, individuals with high hsCRP or low HDL-C (and thus potentially high CHR) might be more likely to be on statins, which could influence CMM risk independently. Subsequent investigations should focus on the comprehensive collection and analysis of relevant data.

## Conclusion

The present study revealed a significant association between elevated CHR levels and an increased risk of CMM. Our findings indicate that CHR, when considered alongside other risk factors, could serve as a valuable biomarker for identifying individuals at heightened risk of developing CMM. In clinical practice, it is imperative to establish CVD risk assessment standards that incorporate CHR and to encourage clinicians to include CHR measurement in routine examinations to enable the early identification of high-risk populations. It is advisable to develop personalized treatment strategies based on the patient’s CHR level and other risk factors, such as hypertension, kidney disease, smoking status, alcohol consumption, rural residence, marital status, educational attainment, WBC, PLT, and hemoglobin levels. These strategies should integrate both pharmacological interventions and lifestyle modifications to reduce the risk of cardiovascular disease.

## Data Availability

The original contributions presented in the study are included in the article/Supplementary material, further inquiries can be directed to the corresponding author.

## Footnotes

^1^ http://charls.pku.edu.cn/
